# Working at a remove: continuous, collective, and configurative approaches to qualitative secondary analysis

**DOI:** 10.1007/s11135-021-01105-x

**Published:** 2021-03-16

**Authors:** Kahryn Hughes, Jason Hughes, Anna Tarrant

**Affiliations:** 1 Sociology and Social Policy, University of Leeds, 11.24 Social Sciences Building, Leeds, LS2 9JT UK; 2 School of Media, Communications and Sociology, University of Leicester, 1.06 107-111 Princess Road East, Leicester, UK; 3 School of Social and Political Sciences, University of Lincoln, 3208 Bridge House, Lincoln, LN6 7TS UK

**Keywords:** Continuous, Collective and configurative QSA, Proximity and distance, Qualitative Secondary Analysis, Recontextualisation and reconnection, Timescapes

## Abstract

In this paper, we define and operationalise three modes of research engagement using qualitative secondary analysis (QSA). We characterise these forms of engagement as *continuous*, *collective* and *configurative. Continuous* QSA involves modes of engagement that centre on asking new questions of existing datasets to (re)apprehend empirical evidence, and develop continuous (or contiguous) samples in ways that principally leverage epistemic distance. *Collective* QSA characteristically involves generating dialogue between members of different research teams to establish comparisons and linkages across studies, and formulate new analytic directions harnessing relational distance. *Configurative* QSA refers to how existing data are brought into conversation with broader sources of theory and evidence, typically in ways which exploit greater temporal distance. In relation to each mode of engagement we discuss how processes of both (*re)contextualisation* and (*re)connection* offer opportunities for new analytical engagement through different combinations and degrees of proximity to, and distance from, the formative contexts of data production.

## Introduction

The ongoing revolution in the digital data landscape has given rise to a vast international network of research data repositories and infrastructures (Corti et al. 2016; Hughes and Tarrant 2020a; Edwards et al. 2020). Together these present hitherto unparalleled opportunities for research data reuse, methodological innovation, and new modes of research engagement (Mason 2007; Bishop 2007, 2009; Neale 2013; Edwards et al 2020). These repositories may contain both qualitative and quantitative research data. While there are well-established methods and approaches for the reuse of quantitative research data, debates on appropriate methods for reusing *qualitative* research data suggest this enterprise is far from straightforward. In particular, the contextualised character of qualitative data production requires considerable theoretical work by researchers seeking to reuse them (Mauthner et al. 1998; Goodwin and O’Connor 2006; Moore 2007; Geiger et al. 2010; Irwin and Winterton 2012c; Tarrant and Hughes 2019; Lyon and Crow 2020). This complexity has driven a growing body of work on the distinctive affordances and defining challenges of qualitative secondary analysis (QSA) (Bishop 2009; O’Connor and Goodwin 2010; Irwin and Winterton 2011a, b, 2012a, b; Bornat et al. 2012; Davidson et al. 2018; Tarrant and Hughes 2019; Hughes et al. 2020b).

There is a long tradition of work addressing questions concerning how researchers might return to research data at varying degrees of ‘remove’, and allied debates about data reuse. In addition to the QSA work already cited, debates surrounding working at a temporal remove, for example, have been pivotal in the development of qualitative longitudinal (QL) research methodologies (e.g. Thomson et al. 2003; Henderson et al. 2012; Holland et al. 2006; Neale 2019). However, rather than viewing such concerns as the exclusive domain of discrete and specialist methodological fields, our arguments in this paper proceed from viewing *all* qualitative research as having ‘secondary’, ‘longitudinal’, and ‘reuse’ components. Such aspects of qualitative research may sometimes be treated explicitly and self-consciously (Thomson and McLeod 2015), and sometimes in ways that are scarcely acknowledged.

From our standpoint, all qualitative research involves temporal frames spanning different timescales. These range from, say, the beginning of an interview to its end; or, for example, from the development of theoretical precepts employed in a study long before that study’s inception; and/or extending beyond the publication of findings from a piece of research, where such findings have yielded insights for scholars several decades later (e.g., Bornat et al. 2012; Hughes et al. 2020b; see also Bishop and Kuula-Luumi 2017). Similarly, all qualitative research involves varying degrees of ‘secondary’ analytical remove, including, for instance, the different levels of involvement in primary data generation of members of the same research team (Frost et al. 2010; Thomson et al. 2012; Phoenix et al. 2016); or when those same members revisit data at varying times after these were originally produced (see, for a fuller account of this position, Hughes et al. 2020b).

In this sense, questions of ‘remove’ are pertinent to all forms of qualitative research. In the case of QSA, however, this is the defining concern. Our aim in this paper is to reframe questions of remove in QSA as relating to how best to take account of, and indeed harness, both the analytical limitations and affordances of varying degrees of temporal, relational and epistemic ‘proximity’ and ‘distance’ from the formative contexts of data production (Hughes et al. 2020b).

Accordingly, we systematise three approaches to how this might be undertaken in relation to three modes of QSA engagement: *continuous*, *collective*, and *configurative*. We present these three modes of research engagement via QSA as an heuristic, each foregrounding the analytical affordances of particular kinds of remove: respectively, epistemic, relational, and temporal (see also Table 2 below). We illustrate each mode of engagement via a series of empirical QSA worked examples. These show some, but by no means all, of the discrete analytical possibilities that various kinds of remove might present via QSA. They also highlight how different modes of engagement inevitably flow into and overlap with each other in ways that can be productively combined and consciously integrated.

The paper builds upon our work elsewhere (see Tarrant and Hughes 2019, 2020a, b; Hughes and Tarrant 2020a, b; Hughes et al. 2020b) by developing and expanding two central lines of argument. First, through our directly challenging the notion that working at varying degrees of remove from formative data contexts is exclusively a source of empirical and analytic deficit (Irwin and Winterton 2011a; Hughes et al. 2020b). Here we explore precisely how ‘remove’ and ‘distance’ can, indeed, serve as the basis for distinctive kinds of insight and may be alloyed and blended with more ‘proximal’ insights through particular modes of QSA research engagement. Relatedly, our second line of argument is that QSA is not simply a question of ‘analysis’ in the narrower sense of ‘reductive’ techniques or procedures; rather, that it involves modes of research *engagement* in the round. In this way, we move from a view of data as a neutral and reified ‘product’, towards a consideration of how researchers *apprehend* different orders of data to recast these as *evidence* (Hughes et al. 2020b). Moreover, we propose that such engagement can be more ‘synthetic’, entailing not just the (*re)contextualisation* but also the (*re)connection* of research through QSA, as researchers repurpose data in new ways and in new contexts (Moore 2007). Such reconnection in part involves the synthesis of different orders of data by bringing them into conversation with one another, as well as with evidence, findings, theory, and developments in the social world beyond the original study contexts (see also, Irwin and Winterton 2011a; Fielding and Fielding 2000).^1^

## Background

Early debate on whether it was possible to re-use qualitative data emphasised the distinction between primary and secondary analysts based on their connections to, or remove from, the contexts of data production (Mauthner et al. 1998; Mauthner and Parry 2010). This distinction had consequences for the very language used to describe what it is we are doing when analysing data gathered in previous research, and perhaps by other teams. The work of Mauthner et al. (1998) was particularly useful in showing how direct researcher experience can inform on data analysis in ways which secondary analysis cannot. As debate continued, however, the rigidity of the distinction between primary and secondary analysts was increasingly called into question (Moore 2007). For instance, to talk about ‘reusing’ ‘pre-existing data’ detracts from how data are always co-produced (Moore 2007) and (re)produced in new contexts. Accordingly, (re)using data can be understood to involve primary analysis of a different order of data (Moore 2007; see also Henderson et al. 2006; Hughes et al. 2016, 2020b).

Building on Moore’s work, we have elsewhere sought to recast a distinction between primary and secondary analysis in terms of a consideration of the different *degrees* and *characters* of ‘proximity’ and ‘distance’ from the formative contexts of data production (see, in particular, Hughes et al. 2020b). Doing so entails a further move away from binary distinctions between primary and secondary analysis, analysts, or data, and instead involves careful and precise examination and articulation of researchers’ relationships with the data with which they are engaged. In QSA, this includes, crucially, a critical engagement with the ‘embedded contexts’ (Irwin et al. 2012) of previous studies as well as with the contexts of subsequent researchers working to make sense of existing data and their usefulness (Bornat et al. 2008; Hughes and Tarrant 2020a, b). Thus, QSA necessitates reflexive engagement with how we may bring differently constituted data into analytic conversation and alignment (Irwin 2013), how this involves recasting them as theoretical objects (Tarrant 2017), identifying not only how they were produced, but how and why they may be (re)used (see also Hughes et al. 2016).

Furthermore, this reflexive engagement through QSA involves not only (re)contextualisation (Moore 2007), but also forms of (re)connection wherein researchers engage with questions of how data are conceived as part and parcel of the context of their becoming. Consequently, contexts are here understood not as fixed, separate, exterior, containers wherein data are somehow located but as the dynamic relational nexuses of the broader social worlds of which they form an integral part. As such, data are neither ‘fixed’ in any simple sense, nor can they ever be entirely separated or disconnected from the contexts of their production (see also Savage 2010).

We have found it useful to set out key activities involved in QSA in terms of (re)contextualisation and (re)connection in order to clarify how reflexive engagement might be supported in QSA. (Re)contextualisation typically encourages us to engage in a reductive, analytical, process through which to explicate degrees of proximity and distance from a particular set of data. This includes considering how data are specific to or located within particular ‘contexts’, such as the formative contexts of their becoming, and how, accordingly they might speak only to and of those contexts. By contrast, (re)connection foregrounds a more integrative, synthetic, mode of engagement that considers how (and which) data might be used to speak beyond the contexts of their generation (see Hughes et al. 2020b). Synthetic (re)connection emphasises connections, emergences, and various other levels of integration that cannot entirely be understood in isolation or through disconnection. Here, processes of (re)connection might entail examining lineages of both continuity and discontinuity between different kinds of research engagement, contemporaneous and subsequent to an original study. More concretely, these differences between (re)contextualisation (e.g. how data express aspects of the contexts of their becoming) and (re)connection (e.g. how data might be used to speak beyond such contexts) underpin different kinds of reflexive engagement with data in the practice of QSA. Table 1 provides a summary of how such modes of engagement might be expressed as concrete questions when researchers undertake QSA. This is not an exhaustive list, but is indicative of key concerns underpinning how far and in what ways it is possible for researchers to repurpose data produced in different formative research contexts, as well as identifying both the limitations to and affordances of such work.

**Table 1:** (Re)contextualisation and (Re)connection as questions about existing data

(Re)contextualisation	(Re)connection
How are these data of their time and place?	What are our connections to the data and the contexts of their production?
How might these data speak both to and of the social contexts of their becoming?	How might these data be re/used to build a new study and/or generate new findings?
To what extent can these data be used to speak beyond the contexts of their becoming, and for what purposes?	How might the datasets need to be developed, extended, augmented and/or recast to support and develop new analyses?
What are the limits to re/apprehending these data and recasting them as other orders of evidence?	How might distance from the contexts of the original production of these data be analytically leveraged?
How are our ‘freedoms to tell’ about the social world curtailed by contextual specifics?	What new ‘freedoms to tell’ are made possible by remove from the contexts of data production?

These processes of (re)contextualisation and (re)connection serve to attune researchers to questions pertaining to the kinds of remove involved in QSA both in terms of the analytical affordances (what becomes possible) and deficits (the limitations of possibility). Rather than involving researchers in practices of classification (e.g. of definitional distinctions between ‘primary’ and ‘secondary’ research) then, these questions instead steer researchers towards substantive, reflexive, analytical and synthetic work germane to their particular study and the modes of (re)engagement undertaken. In turn, this process of elucidating and articulating the degrees and character of proximity and distance from the formative contexts of data production enables a consideration both of the limitations to, as well as the distinctive analytical affordances of, research engagement via QSA.

We have elsewhere (Hughes et al. 2020b) made the case that ‘being there’—involved directly in the formative generation of data—offers distinctive insights potentially not recoverable through ‘secondary’ analysis. Tacit experiential and ‘felt’ understanding, sensory perceptions, participation in what we might describe as knowledge collectives are integral to formative research contexts. Attention to data as something that researchers, participants, and others ‘do’ through particular kinds of engagement highlights the social dynamics of research encounters. Nonetheless, the insights drawn from direct involvement in such encounters can, at least in part, be communicated through research outputs (see Irwin and Winterton 2011a; Irwin 2013; Neale 2019). Moreover, varying degrees of ‘remove’ provide opportunities for other kinds of insight, engagement and analytical apprehension. This is particularly so, when proximal insights are brought into conversation with subsequent sources of evidence, research, theory and developments in the social world (Corti et al. 2005; Andrews 2013; Hughes et al. 2020b). Below, we outline more concretely how researchers might undertake such ‘conversations’ between more proximal and distal insights, and between theory and evidence more generally, through three modes of research engagement via QSA.

## Continuous, collective, and configurative QSA

In series, each form of engagement typically, but not *necessarily*, describes progressively greater degrees of ‘remove’ from the formative contexts of data production and relatedly different strategies for (re)contextualisation and (re)connection. They each overlap considerably but are nonetheless delineated here for heuristic purposes to capture something of their distinctive emphases—respectively, *epistemic*, *relational*, and *temporal* distance.

The first of these is *continuous*. Continuous research engagement might involve using QSA variously to compare, contrast, and case project data sets for the purposes of identifying analytic correspondences and distinctions (Tarrant and Hughes 2020a, b). It might also entail QSA researchers considering new questions, identifying and generating new empirical evidence, and for instance, expanding datasets to build continuous (that is, temporally and relationally contiguous) samples (Irwin and Winterton 2011a; Tarrant 2017). Here, the ‘analytic conversation’ involves researchers asking new kinds of questions of existing research, as well as using existing data to support the formulation of new questions for new research.

This mode of QSA is characteristically temporally continuous. That is, it involves research and data that continue over time in a connected manner (for example, extending to a second phase of a study, scaling up or out of an existing piece of research, and so forth). It is also often relationally continuous, that is, offering researchers access to existing relational networks, perhaps even under similar, social and institutional conditions. The principal source of greater ‘remove’ from formative data production foregrounded by continuous QSA is ‘epistemic’—whereby researchers are engaged in asking new questions of data in ways that might necessitate expanding and/or extending data sets, and/or re-apprehending them, in particular kinds of ways (Tarrant 2017).

Our second mode of research engagement is *collective* QSA. Here, ‘relational’ distance is consciously harnessed. For instance, members of different research teams from similar, cognate or even entirely distinctive projects and the broader relational nexuses that these entail, are purposefully brought together in order to permit a dialogue of evidence and questioning, for example, through theoretically sampling from across datasets. In collective QSA, researchers might explore the extent to which and how datasets may be analytically linked, aligned, distinguished, contrasted, sometimes for the purposes of corroboration, empirical extension, and familiarisation for scholars new to those datasets. At other times, collective QSA might be developed in order to consider the possibilities for new analytic directions (Bornat et al. 2012; Tarrant and Hughes 2020a, b).

Such new directions may involve retaining the epistemic priorities of the original research, but complementing and augmenting the analyses undertaken through the greater comparative and dialogic opportunities made possible through collective QSA. Here the analytic ‘conversation’ occurs between members of a research team with one another and with members of other research teams. It is also a conversation of evidence, theory, and findings from beyond the immediate contexts of the original study. The principal source of distance leveraged in the case of collective research engagement via QSA is ‘relational’. That is, it involves, consciously and intentionally broadening the relational nexuses of the research through direct (and sometimes indirect) dialogue with researchers in similar, cognate, and sometimes different areas. Such collective analytic conversations are characteristically two-way, perhaps multi-directional. For instance, just as we might develop new insights through considering other researchers’ findings, evidence, theories, and so forth, so they might from ours with, moreover, entirely different interpretations of our data—a view of these ‘from a distance’ that yields important new insights. Such collective analytic conversations through QSA might be face-to-face, via organised workshops, or more distal, perhaps asynchronous, at a physical and spatial remove.

The third, *configurative* mode of research engagement via QSA, foregrounds questions of how research and data form part and parcel of the social world they are being used to ‘tell about’ (Becker 2007). Here a combination of all three forms of distance (epistemic, relational, and temporal) will often be involved, but typically at a greater *temporal* remove, that is, at a time *significantly after* the original research. That phrase ‘significantly after’, not simply ‘later’, is here intended to highlight how what constitutes a temporally significant span of time is an empirically germane question irreducible to arbitrary normative conceptions of time (e.g. ‘a few years later’, ‘a decade later’, ‘further down the line’, etc.). For example, a significant timespan in a study that seeks to document changing attitudes towards gender differences might involve a very different chronology from that of, say, a dataset charting responses to the Covid-19 pandemic. Configurative QSA typically involves subsequent researchers, sometimes those with no direct involvement in the formative contexts of data production, revisiting existing datasets and recasting these, re-apprehending these, as forms of evidence that can be used both to tell about themselves and the broader social *figurations* of which they form part (Elias 2012; Hughes et al. 2016), including data not previously considered to constitute evidence (O’Connor and Goodwin 2017).

Characteristically, configurative research engagement via QSA involves researchers bringing an existing dataset, or indeed datasets, into conversation with evidence, theory, research findings, and other social developments subsequent to its/their initial collation and development (Gillies and Edwards 2012). Examples of configurative QSA vary considerably. They include, for instance, reconsidering the evidence of a study that ostensibly failed to meet its espoused objectives in order to explore what insights such ‘failure’ might yield in the light of subsequent developments (see, for example, Tarrant and Hughes 2019). Configurative QSA might also involve reimagining data in terms of how, for example, it may speak to its own historical juncture. How, for instance, a study of obedience to authority undertaken in the 1960s can be used to speak to prevailing ethical safeguards in research institutions of the time, or, for example, how the pursuit of concerns in post-war US social psychological research expressed concerns about the particular conditions that made possible the holocaust.^2^

Below we present these three forms of QSA engagement in relation to empirical illustrations of each. The different studies we discuss below are offered not so much as exemplars, but as worked examples. As examples, these are at the ‘proximal’ end (epistemically, relationally, and temporally) of each mode of working at a distance, and as such ‘within the reach’ of what QSA might conventionally be understood to comprise.^3^ While we describe them sequentially, we suggest these modes variously imply and overlap one another both in approach and in terms of their temporal sequencing. We also propose these three modes of QSA have broader application than we have used them here, do not need to be used in this order, can be used in any combination, and may be fruitful when used independently.

## Continuous qualitative secondary analysis^4^

Our illustration of c*ontinuous* QSA uses the example of Tarrant’s Leverhulme Fellowship work entitled *Men, Poverty and Lifetimes of Care* (henceforth MPLC) which involved the (re)use of two studies. The first of these studies was the 2007–2012 *Intergenerational Exchange: Mid-life Grandparents and HealthInequalities* programme (henceforth Grandparenting) exploring the longitudinal experiences, identities and health needs of mid-life grandparents aged 35–55 years in the north east of England (Hughes and Emmel 2012). In the Grandparenting study, mid-life grandparents were interviewed across four waves of data collection. The second study drawn upon in Tarrant’s Fellowship work was the 2012–2015 *Following Young Fathers* programme (henceforth Young Fathers) (Neale et al. 2015). This study focused on the longitudinal experiences, identities and support needs of young fathers, aged 25 and under, and involved five waves of interviews, also in the north east of England. The Grandparenting and Young Fathers studies had certain key continuities: both had their bases in the *Timescapes: Changing Lives and Times* programme of research (Neale and Holland, 2007–2012), were conducted in the same UK city, and included people who lived in similar localities.

Tarrant undertook continuous QSA utilising these two studies for the distinctive purpose of developing both the research questions and the research design of MPLC. Here, she centrally explored the potential for the Grandparenting and Young Fathers studies to offer new insights into the complex sets of intergenerational responsibilities, relationships and hardships that the grandfathers and teenage fathers in the studies navigate. These substantive areas of concern were not considered by the original research teams, and thus constitute the principal source of Tarrant’s epistemic departure.

### (Re)contextualisation

Tarrant commenced her QSA work by engaging in a process of recontextualisation which initially involved familiarisation with the data and outputs from both studies, including learning about the study histories and the contextual specifics of their becoming through conversations with researchers from the original teams. These preliminary analyses identified empirical content relating to men’s longitudinal and intergenerational experiences of caring responsibilities. Further exploration involved an ongoing, often recursive, process of theoretical sampling for ‘emblematic cases’ (Thomson et al. 2014) across both datasets. The new evidence of men’s caring roles and responsibilities in low-income contexts identified through Tarrant’s provisional analytical work had hitherto been missing or obscured in research and policy on low-income family life, in ways not identified by either of the original research teams (Tarrant 2017; Tarrant and Hughes 2019). Such considerations were not so much neglected by the original research teams, but rather, were secondary to their epistemic priorities, which centred on elucidating specific generational identities. Here, then, the process of recontextualisation undertaken by Author C identified the epistemic priorities of the original research (including how these were expressive of a particular social, intellectual and policy milieu) which, in turn, enabled a consideration of how the data might be repurposed, through a process of (re)connection, for a new study of men’s caring in low-income contexts.

### (Re)connection

(Re)connection involved developing further lines of enquiry in MPLC to generate new empirical evidence with an extended sample of young fathers, mid-life fathers and grandfathers, some of whom were directly drawn from the Young Fathers study (Tarrant 2021). This wider sample was based on the recognition that men hold multiple generational identities in different generational positions (Tarrant and Hughes 2019). (Re)connection via continuous QSA involved Tarrant taking themes, participant samples, and essential gatekeeper/stakeholder relationships forward through time in an ongoing research process. By building on relationships with a number of the original project partners, Tarrant thereby gained an extended insight into the changing policy contexts of the localities in which the original studies were conducted, as well as access to original participants (Tarrant 2017). The relatively close timeframes of all three studies enabled her to carry forward a whole range of practical, yet valuable resources, in the form of relationships, connections and professional recognition in MPLC (Tarrant 2017; Tarrant 2021). The timeframes and the scope of the MPLC research were subsequently elongated and enhanced in a number of directions. Continuity was also supported through her building new samples contiguous to those from the previous studies, thus extending the temporal reach of the original datasets through complementary research in similar localities.

As this worked example serves to demonstrate, continuous QSA advances existing scholarship via epistemic ‘distance’ in the following ways. First, it allows for the development of new empirically-informed research and agendas, where questions drawn from academic debate can be brought into direct conversation with new insights from existing data in preparation for further empirical work. It allows researchers to identify the limitations of datasets in the context of new questions, and supports the identification of what new data are required to answer specific questions more fully. An additional strength of this approach is where such insights and new potential evidence ‘developed at a distance’ can be blended and alloyed with that of original studies through retaining certain epistemic continuities, here in relation to a focus on low-income contexts and the longitudinal significance of family involvement for men. Furthermore, continuous QSA allows research to build directly upon valuable connections with the original research team, and, as was the case for Tarrant’s research, enables the further utilisation and development of key gatekeeper and stakeholder relationships.

## Collective qualitative secondary analysis

Our second worked example, here of *collective* QSA, draws on a two-day workshop of which Author A was a part, which involved bringing together the research teams from two separate studies, again under *Timescapes: *the Grandparenting study (as described above) and The OldestGeneration (TOG).^5^ This workshop was held to explore and promote methods and strategies for reusing archived QL data such as through collaborative analysis (see especially Bornat et al. 2008; also, Irwin et al. 2012; Thomson et al. 2012; Haynes and Jones 2012). Indeed, our model of *collective* QSA^6^ builds directly out of this tranche of work. An explicit focus of the workshop was to consider the possibilities of sharing particular participants across both studies for the purposes of broadening the study samples according to characteristics of age and participants’ socio-economic circumstances. An additional aim was to consider how far interview narratives from participants from ostensibly similar socio-economic contexts might be brought together for cross-study comparison and, perhaps, connection.

The two studies appeared to have obvious connections. The Grandparenting study looked explicitly at what grandparents did for their grandchildren, and TOG researched people over the age of 75 about changing family life, who might be expected to have some experience of grandparenting. Both studies were qualitative and longitudinal; they explored family changes over time, and developed detailed intergenerational retrospectives of personal and family lives with their respective participants.

The workshop itself is best understood as part of a longer process of exchanges (principally via email) between the Grandparenting and TOG research teams. These took place for several weeks before the workshop, allowing for the development of key questions for reflection, an agenda, and for other preparatory work to be agreed. The exchanges also continued sporadically for several years after the workshops were held. The central aim was to generate a dialogue between the relationally more proximal and distal insights of members of the Grandparenting and TOG research teams.^7^

Accordingly, three key objectives were agreed between the teams. The first was to explore the possibilities for emergent and interpretive analysis of each other’s data and the potential for thematic exchange and development. The second was to develop and then to address a set of thematically-driven questions concerning grandparenting—a concern provisionally assumed to constitute a key point of empirical intersection between the two studies—that would help refine and extend the questions either team might address in their own analyses. The third was to consider the methodological possibilities for, and challenges to, sample boosting via the direct inclusion of one or more cases from each other’s study.

### Recontextualisation

The preparatory work for the workshop included an early aspect of recontextualisation which involved researchers individually re-approaching their own datasets to identify cases that spoke directly to the new thematic and methodological questions derived from the early dialogue between research teams. Accordingly, two cases were selected from the Grandparenting study and three from TOG, and forwarded to the other team. This process of intensive casing (Emmel and Hughes 2009; Henderson et al. 2012) thus required researchers to view both their own data (through considering related but new questions), and the other team’s data, from a greater ‘distance’.

The recontextualisation phase of this collective QSA continued in the early discussions at the workshop. This involved prompting individual researchers to ‘find themselves in their data’. That is to say, team members were steered towards reflecting upon their own involvements in the production of the datasets from their study, the emergent character of the contexts of data production, and their particular imprints on the data produced. In turn, such reflections were compared so as to yield insights into how each dataset expressed certain of the epistemic, relational, and temporal conditions of its becoming.

This experiential dialogue yielded manifold observations. For instance, there were marked differences between the researchers of either team with respect to how far they questioned or probed what was said by participants at interview. Reflection on such differences clarified that while researchers in both studies used a life-history approach, those in the Grandparenting study tended towards tracking across extensive ‘family cases’, while those in TOG developed rich oral histories. Further, empirical differences between participant samples shaped interview techniques: in TOG there was a greater sensitivity (and related practice) towards critically probing what was said at interview in order to develop more comprehensive and detailed accounts of the extended life histories of the participants and their families. In the Grandparenting study, there was more caution around the use of particular policy-related language and terminology that commonly saturated participants everyday involvements with health and social care services. This was driven by an experiential recognition within the research team of how participants in low-income contexts utilised policy language in leveraging access to essential resources. Also, these participants were often forced to resist or contest policy terms in order to avoid potentially punitive measures or further stigmatisation (see also Ridge 2009; Neale and Clayton 2014; Wright and Patrick 2019; Tarrant and Hughes 2019).

Such differences in interviewing techniques additionally expressed varying ethical sensitivities and strategies. In the Grandparenting study,, there was a shared concern that probing follow-up questions might distress participants, and a related sense that silences, or the sometimes pointed closing down of discussions by participants of particular topics at specific response junctures, may speak to incidences of abuse and abusive relationships narrated in relation to other aspects of participants’ lives (see also Bornat et al. 2008; Irwin et al. 2012). In TOG there was a much greater emphasis on the sensitive negotiation of such ethical concerns at the beginning of each interview, checking that interviewees were aware of what had been agreed, the aims of the research, the kinds of question that would be asked, and how participants might deal with questions that made them feel uncomfortable. Consequently, therefore, the discussion of seemingly modest differences in approach and method opened the way for the development of more wide-reaching observations. In particular, some relating to precisely how the data and the research teams’ generation of them in either study was simultaneously contextually and situationally contingent, and empirically and analytically germane (see also Hughes et al. 2020b).

This process of ‘finding the self in the data’ proved difficult to disentangle from the finding of other aspects of the ‘context’ of data production. For instance, the timeframes and time-horizons expressed in the interview discussions, both by participants and interviewers, contrasted significantly between the two studies. Such differences in temporal sensitivity were found to speak beyond epistemic priorities and researcher sensitivities. In the Grandparenting study, the interviewees’ lives were characterised by vulnerability to ‘shocks’ and ‘tipping points’ into chaos (Emmel and Hughes 2010). Their narratives often focused on events at some remove historically, but which nevertheless were used to express stubborn and persistent experiences of marginalisation and poverty. These participants were able to provide detailed family histories but their ‘future orientations’ were often constrained to the extent that they could only think about ‘one day at a time.’ Additionally, their lives were characterised by significant deprivation and ongoing efforts to ‘make ends meet’, and these aspects of their lives often orientated their accounts towards a ‘continuous present’ (Emmel and Hughes 2014). The TOG interviewees were much older, typically in more secure and resourced situations, expressed through their ability to reflect back several decades to earlier times when they were children or young parents. In this way, the workshops yielded considerable insights into the intersections between timescapes (Adam 1998), timescales (see also Bornat and Bytheway 2012) and socio-economic differences.

Through these and related discussions, it emerged that while combined the two datasets facilitated a comprehensive overview of a broad range of grandparenting experiences, identities and practices, there was little overlap of the everyday experiences of grandparenting among participants across the two studies. Because of differences in the sampling criteria as they related to age in the two studies, there was no possibility for sample boosting via the direct inclusion of one or more cases from the other study.^8^

### (Re)connection

Despite these major differences, the two projects *when brought into conversation* were found to present new possibilities for (re)connection. Significant insights were generated, for example, about the rich possibilities for reconnection to the original studies through more nuanced gender analyses of the IGE data. For instance, how an understanding of the longitudinal dynamics of men’s relationships with women could not be generated solely through recourse to narratives of family relationships. The analytical and evidential conversations instead suggested that, crucially, such work required broader theoretical conceptions of place. Among the manifold insights and observations yielded relating to men’s contributions to the family, the workshop discussions accordingly signposted analytic directions concerning how and through which relationships (e.g. as sons, fathers, husbands) men supported, or otherwise, women in family contexts. While such signposts richly informed the ongoing analyses of the IGE team, ultimately, the workshops uncovered that the proper pursuit of such lines of analysis necessitated the development of new data and/or new lines of enquiry.

As such, processes of (re)contextualisation and (re)connection in this example together highlighted the limits to (and in one case, the impossibility of) meeting some of the key objectives developed in the early exchanges between the Grandparenting and TOG teams. A divergence in samples and data constrained by the substantive focus of each meant particular lines of enquiry, and the potential for direct sample sharing, were not possible. Yet what emerged instead were analytical possibilities and future directions developed through a dialogue of relationally proximal and more distal insights and observations. These directions, this ‘outcome’, not anticipated at the outset of the workshops, subsequently came to underpin further research that would otherwise not have been realised. Indeed, in conversations some five years later at a three-day workshop on intergenerational exchange in Prague, Hughes K and Tarrant discussed the workshop findings in relation to Tarrant’s own research interests, beginning 6 years of further collaboration.

## Configurative QSA^9^

Our recent QSA work relating to a study of problem internet gambling (Hughes et al. 2020b) serves as a worked example of configurative QSA. The original study, funded by the Economic and Social Research Council (ESRC) and Research in Gambling Trust (RiGT), analysed the impact of problem internet gambling on the family, generating a total of 69 interviews from successive waves of data collection (Valentine and Hughes 2006–2008). Hughes was directly involved in this original research, and also our QSA of the data it generated some 12 years later. In the recent 2020 study, we (re)used the data for purposes markedly different from those of the original study. Here, our subsequent QSA work involved a definitive epistemic break through a new focus upon how we might reapprehend the data to speak to debates concerning the character of interview talk; the modes of participation, engagement and questioning that participants bring to interview; and the balance between narrative production and cultural reproduction in interview encounters. Put simply, we reused what we was available of the original dataset to work through specific methodological questions about how far the form of an interview shaped analyses of its content. Accordingly, we centrally harnessed all three forms of ‘distance’ from the original study — epistemic, relational, and, in particular, temporal — with a focus on how these inter-related in order to develop new understandings of the data through a dialogue between distal insights (particularly those made possible by a decade of temporal remove) and more proximal insights, in particular, those derived from Hughes K’s direct involvement in the original research and its published outcomes.

### (Re)contextualisation

The recontextualisation work for this QSA began many years ago through ongoing conversations between the authors concerning Hughes K’s work on how far theoretical development might be supported by, or integral to, methodological reflexivity. In their discussions, Hughes K frequently employed the example of the internet gambling study, referring in particular to an un-investigated aspect of the data relating to participants’ accounts of why they had taken part in the research. Participants in the ESRC/RiGT study when interviewed about their gambling behaviour consistently tended to approach, and in some cases quite consciously construe and narratively express, research interviews as therapeutic encounters. Hughes K’s central question was why? Was this tendency simply expressive of how the ESRC/RiGT study was conducted? Or might the tendency express something about interviews more generally? Might it, indeed, be understood to speak of and to aspects of the ‘interview society’ (Atkinson and Silverman 1997) and of the particular kinds of biographical and narrative work characteristically undertaken in interviews? Put simply, these questions can be understood as variations of our pivotal recontextualisation questions: to what extent are these data expressive of the contexts of their becoming? And: can these data be used to speak beyond themselves? Such questions were shared and discussed through several research collaborations between the various authors, and they became central to the 2020 QSA project (Hughes et al. 2020b; see also Hughes J et al. 2020a) (Table 2).

**Table 2:** The three ‘C’s of QSA—an heuristic summary

	Remove	(Re)contextualisation focus	(Re)connection focus
Continuous	Foregrounds epistemic remove:	(Re)familiarisation with the data by researcher(s) Identifying epistemic priorities and specifics of original research Exploring the extent to which and how data might be repurposed/recasted as evidence through further extension and development	Asking new questions of the data, bringing them into conversation with new debates and evidence Continuing and extending research through time: generating new data to speak to new epistemic priorities Blending/combining and comparing insights at an epistemic distance from those drawn from original study
Collective	Foregrounds relational remove: different research teams, different relationships to data	Re-appraising relationships with data Researchers look to ‘find themselves and one another in their data’ through a dialogue of simultaneously individual and collective forms of ‘reflexivity’ Different teams of researchers identify points of overlap, intersection and distinction in one another’s data	Data from different projects and studies brought into conversation by research teams Different research teams collectively highlight how one another’s data might serve as the basis for new lines of enquiry, or might be extended through the development of new data Researchers seek to synthesise insights harnessing relational distance
Configurative	Foregrounds interplay between temporal, epistemic, and relational remove	Researchers consider how data might be used to ‘tell about’ both the specific contexts of their becoming and the broader social ‘figurations’ of which these formed part A central focus on the interrelationship between the kinds of questions asked by specific groups of researchers and the social conditions under which these epistemic priorities developed Data approached simultaneously as ‘subject’ and ‘object’ of (re)engagement: how data are ‘of their time’ and how they might be used to speak to developments since (and before) their generation	Data from a previous study brought into ‘configurative’ conversation with subsequent developments in the social world, new scholarship and evidence, new theories and debates Researchers reapproach data through redrawing lines of epistemic, relational and temporal continuity and discontinuity Researchers recast data as new forms of evidence to revisit debates from the time the original research was conducted, and to speak to issues and concerns as they have developed since then

In preparation for our QSA, Hughes K developed a file of extracts drawn from a partial sample of the ESRC/RiGT study. The dataset was partial because not all the data had been shared during the original study, and over-zealous data protection work caused a central, shared file to be deleted. While Hughes K had all the transcripts in paper form, she had no resources for their digitisation, and so only 28 out of a possible 69 interviews with gamblers and their significant others were available. However, these were extensive, and all contained transcriptions of participants’ responses to questions about why they had taken part in the research. Hughes K extended the extracts to include much larger sections of the preceding and following interview talk around these responses. These sections were found to include questions by participants, particularly around how the research team understood internet gambling, what the purposes of the research was, who was it for, and what the team had discovered from other participants. The complete transcripts were then searched for any other questions asked by participants, and additional data extracts were added to the larger file. On this basis, whole transcripts were selected for depth analyses which were multi-directional, and multi-modular (Tarrant and Hughes 2020a, b).

Consistent with our aim to undertake a ‘synthetic’ (re)engagement with the data from the original study, the authors brought each of their individual analyses of the interview data into collective conversation, exploring in particular differences between Hughes J and Tarrant’s relationally more distal observations and insights (through their having not been involved in the original study) and those of Hughes K who had been part of the original team. This centrally involved a consideration of how the data from the original study could be understood as characteristic of the time, place, and conditions of their becoming. Accordingly, a key insight developed through the QSA was how, more than a decade after the original research, the distinction between ‘online’ and ‘offline’ gambling employed by researchers in the set-up of the study could be understood as expressive of a particular phase in the development of internet gambling and, more generally, in processes involving the growing pervasiveness of the internet into everyday life (Hughes et al. 2020b).

### (Re)connection

Part of what emerged from such analytical work was our ‘surprise at the surprise’ of the original research team that internet gamblers included a range of activities—bingo, slots, poker and sports betting among them—in their discussion of their *internet* gambling. Working at the temporal remove of more than a decade, we found it considerably less ‘surprising’ to find this blurring of online and offline activities. From the standpoint of the present, such blurring is commonplace. For example, ‘internet’ gambling might involve participation from a football stadium at a live event, retrieving the odds for the next player to score and placing a bet in real time via a smartphone (Hughes et al. 2020b).

Such observations yielded manifold insights, both about the original study, and about how we might (re)approach the data. Accordingly reconnection, in this case, involved redrawing lines of continuity and discontinuity. It was not simply a process of ‘going back’ or ‘returning’ to the ‘context’ of the original dataset, but involved reflexively attending to three overlapping axes of ‘distance’ and ‘proximity’. Here temporal distance/proximity yielded insights relating to continuities and changes over time: how the data were ‘of their time’ in certain respects (i.e. expressive of a time in which it made sense to more sharply demarcate online and offline activities), but could also speak beyond themselves about processes of change (nascent developments in internet gambling, more fully realised subsequently).

Relational distance/proximity pertained to the relational nexus within which the original study was undertaken and the degrees of involvement and detachment that we as subsequent researchers had from this. For instance, the degree to which we had more or less direct involvement in the original study’s design and data collection, or our greater ‘distance’ from the institutional and broader social conditions under which the data were first generated. Such considerations were particularly significant to our discussions of how participants, in approaching the interviews as therapeutic encounters, themselves could be understood to be responding not simply to the emergent character of the original research—its framing, the particular style of questioning adopted—but to be engaging in ways that spoke to processes and contexts beyond these interview encounters. For instance, such engagements could be understood as at once expressive of broader cultural tropes relating to interviews as confessionals; social conditions in which opportunities to talk about problem internet gambling were curtailed; and discursive tensions playing out in policy contexts. Further, the interviews illustrated how participants reflexively negotiated the limits to the cultural stock of narratives relating to addiction, and how such narratives were both enabling and constraining in ways that were actively negotiated—casting light on more general debates concerning the character of interviewee participation (see also Hughes J et al. 2020a; Hughes et al. 2020b).

Finally, ‘epistemic’ proximity/distance related to the kinds of questions both expressed in, and asked of, the data produced through the original research and the connections, both continuities and differences, to our (re)engagement, (re)apprehension, and (re)use of the data via QSA to tell about participation and interviewing more generally. Our core argument both there and here, is that (re)apprehending the data enabled us to recast them as new kind of evidence that allowed us to speak to debates both from the time the research was conducted (and even a decade before it), and to debates as they have developed over a decade after the study concluded.

## Conclusion

The key argument we have developed in this paper pertains to the particular affordances of QSA when used in *continuous*, *collective* and *configurative* modes of engagement. Each involves various forms of analytic ‘conversation’: from the face-to-face conversations between members of a team with their former and current selves, and between members of different research teams, to the conversations between generations of scholars. Simultaneously, such conversations are conversations of evidence, findings, theory, and developments in the social world. They entail a distinctive mode of social scientific practice where theories and findings are not so much ‘tested’ in a logical or definitive sense, but rather, refined, developed, extended, perhaps entirely reimagined, through the development of a concerted dialogue between theory and evidence (see also Dunning and Hughes 2013).

We have shown how both proximity to and distance from the formative contexts of data generation present their own affordances and, particularly when combined, offer the basis for further insight when pursued through different kinds of research engagement. These three forms of research engagement illustrate and provide a language for the potentialities that epistemic, relational and temporal distance afford via QSA. Further, these three worked examples provide a starting point for scholars interested in harnessing the enormous analytic potential made possible by the accelerating growth in research data infrastructures.

In practice, these different modes overlap considerably, and may, indeed, be fruitfully combined in the same QSA study. Their separation here is intended to serve the purposes of heuristic exposition, highlighting distinctive analytical emphases and possibilities. It is expressly not our intention to suggest that each mode is analytically discrete.

We additionally advance the argument that QSA involves not just processes of recontextualisation but also reconnection. Such arguments are wedded both to the idea that working at a distance involves affordances as well as deficits with regard to the kinds of insights that it makes possible, and to the related idea that QSA can be conceived more synthetically. In using this term, we highlight exploring *both* continuities and discontinuities, proximity and distance, connection and disconnection, from original research, not as a matter of classification, but as an empirical and substantive consideration germane to the particular topics and investigations undertaken.

## Ethical Statement

‘Working at a Remove’ elaborates three strands of Qualitative Secondary Analysis (QSA) engagement, namely ‘Continuous’, ‘Collective’ and ‘Configurative’. ‘Continuous’ QSA used data gathered under the Timescapes Study, and ‘Collective’ QSA reports on data-sharing work under the Timescapes Study. ‘Configurative’ QSA used data from an ESRC/RiGT funded study on the impacts of internet gambling on the family. We can confirm that formal ethics approval was granted to the Timescapes study by the University of Leeds Research Ethics Committee in 2006, prior to the collection of the interview data used for the ‘Continuous’ and ‘Collective’ Qualitative Secondary Analysis work. We can also confirm that the University of Leeds Research Ethics Committee gave ethical approval for the ESRC/RiGT funded study on the impacts of internet gambling on the family in 2007, in which the interview data used for the ‘Configurative’ QSA was collected. These data were collected with participant consents for reuse. The ethical strategies we employed across the three QSA strands elaborated in ‘Working at a Remove’ include: the pseudonymization of all data; double-anonymisation in the re-presentation of data extracts in form of quotations in our paper.

## Footnotes

1 The idea of QSA involving an ‘analytic conversation’ of different sources and orders of evidence has been explored in depth elsewhere (see, in particular, Irwin and Winterton 2011a). Here we build upon and extend this notion by exploring the possibilities for different kinds of analytic conversation which alternatively leverage various combinations of (epistemic, temporal, relational) ‘proximity’ and ‘remove’ through QSA. Our arguments in this respect also relate to a more general model of sociological practice which involves the conscious development of a ‘two-way-traffic’ of concepts and evidence, theory and research, and relatedly, between different researchers, at differing degrees of remove across time and space. Here the conventional distinction between theory and research gives way to a model of research-theorising as constituting the primary vehicle for sociological analysis. In turn, this understanding implies a particular model of how evidence is ‘tested’, or better, how sociological knowledge might come to have greater adequacy as a means of orientation to the fluid and complex social world of which it forms part through intra and intergenerational ‘dialogue’ (see, for a further development of these arguments, Dunning and Hughes 2013).

2 All three modes of research engagement via QSA entail distinctive ‘depth-to-breadth’ approaches to analysis (e.g. from small portions of ‘depth’ data to contexts extending considerably beyond the production of those data). This complements new work elsewhere with similar aims that has developed ‘Big Qual breadth and depth’ QSA from large data ‘corpuses’ to sustained depth analyses (Davidson et al. 2018; Edwards et al. 2020). We would like to express our gratitude to Professor Rosalind Edwards for suggesting this term.

3 We additionally note here that our work builds out of a long connection with *Timescapes: Changing Lives and Times*, where questions of qualitative data re-use and secondary analysis comprised a central strand of empirical engagement (Irwin and Winterton 2011a,b; Bornat et al 2012), as well as using data from the seven Timescapes empirical projects. see also the Timescapes Archive: https://timescapes-archive.leeds.ac.uk.

4 There are certain connotations to the term ‘continuous’ that are useful for our present purposes, others that are less so. We have opted for the term because it highlights continuities (particularly those of a relational and temporal character) between an original study and its re/apprehension via QSA. It is not, however, intended to imply that this mode of QSA is necessarily ‘constant’, or ‘unending’, or entirely without ‘breaks’ or ‘pauses’ in engagement.

5 This workshop was attended by Joanna Bornat, Bill Bytheway (TOG); Hughes and Lou Hemmerman (Grandparenting study) and Bren Neale, discussant.

6 We have opted for ‘collective’ rather than ‘collaborative’ QSA because we wish to leave open the possibility of conversations between more relationally proximal and distal insights and observations that do not pivot on shared intellectual endeavours, particularly those that happen at considerable degrees of remove (temporally, or otherwise).

7 Again, our example here is of two teams which, particularly by virtue of their both being bracketed by *Timescapes*, are towards the ‘proximal’ end of what we might conceive of as a continuum of relational distance. The point is to show that even here, between research teams under similar institutional conditions, pursuing substantively cognate areas of concern, there were considerable possibilities for harnessing relational distance.

8 It is worth noting that the questions developed for these workshops themselves reflected the conditions of their becoming. Had different questions been asked, or different researchers from different teams been present, workshop discussions may have produced different findings on how and to which epistemic or methodological insights on grandparenting these datasets could be used to speak.

9 The term configurative invokes aspects of both continuous (with respect to its harnessing epistemic distance) and collective (with respect to relational distance) QSA, and thus may encompass these modes of QSA whilst also augmenting them through the leveraging of greater temporal distance. The term derives in part from Elias’s concept of ‘figuration’ (Elias 2012) which refers at once to social relationships, social processes, and the structure of changes within these. Central to the term configurative is the idea that data and methods are not somehow ‘separate’ or ‘neutral’ informants on the ‘social world’, but rather, are part and parcel of the social world: both expressive, and partly constitutive, of the social ‘contexts’ of their ‘becoming’.
